# Low doses to the heart in daily practice for treating left-sided breast cancer using accelerated partial-breast irradiation by multicatheter brachytherapy and deep-inspiration breath-hold using a SIB

**DOI:** 10.1007/s00066-023-02047-z

**Published:** 2023-02-24

**Authors:** Stefan Knippen, Sven Schönherr, Michael Schwedas, Tobias Teichmann, Simon Howitz, Matthias Mäurer, Andrea Wittig-Sauerwein, Marciana-Nona Duma

**Affiliations:** 1 Department of Radiotherapy and Radiation Oncology, University Hospital Jena, Friedrich Schiller University, Jena, Germany; 2 Department of Radiation Oncology, Helios Kliniken Schwerin - University Campus of MSH Medical School Hamburg, Schwerin, Germany; 3 Department of Radiation Oncology, SRH Wald-Klinikum Gera, Gera, Germany; 4 MSH Medical School Hamburg, Department for Human Medicine, Hamburg, Germany

**Keywords:** APBI, Breast cancer, Brachytherapy, DIBH, SIB, Simultaneous integrated boost, Heart

## Abstract

**Purpose:**

The aim of this study was to analyze the heart dose for left-sided breast cancer that can be achieved during daily practice in patients treated with multicatheter brachytherapy (MCBT) accelerated partial-breast irradiation (APBI) and deep-inspiration breath-hold (DIBH) whole-breast irradiation (WBI) using a simultaneous integrated tumor bed boost (SIB)—two different concepts which nonetheless share some patient overlap.

**Materials and methods:**

We analyzed the nominal average dose (Dmean) to the heart as well as the biologically effective dose (BED) and the equivalent dose in 2‑Gy fractions (EQD2) for an α/β of 3 in 30 MCBT-APBI patients and 22 patients treated with DIBH plus SIB. For further dosimetric comparison, we contoured the breast planning target volume (PTV) in each of the brachytherapy planning CTs according to the ESTRO guidelines and computed tangential field plans. Mean dose (Dmean), EQD2 Dmean, and BED Dmean for three dosing schemes were calculated: 50 Gy/25 fractions and two hypofractionated regimens, i.e., 40.05 Gy/15 fractions and 26 Gy/5 fractions. Furthermore, we calculated tangential field plans without a boost for the 22 cases treated with SIB with the standard dosing scheme of 40.05 Gy/15 fractions.

**Results:**

MCBT and DIBH radiation therapy both show low-dose exposure of the heart. As expected, hypofractionation leads to sparing of the heart dose. Although MCBT plans were not optimized regarding dose to the heart, Dmean differed significantly between MCBT and DIBH (1.28 Gy vs. 1.91 Gy, *p* < 0.001) in favor of MCBT, even if the Dmean in each group was very low. In MCBT radiation, the PTV–heart distance is significantly associated with the dose to the heart (*p* < 0.001), but it is not in DIBH radiotherapy using SIB.

**Conclusion:**

In daily practice, both DIBH radiation therapy as well as MCBT show a very low heart exposure and may thus reduce long term cardiac morbidity as compared to currently available long-term clinical data of patients treated with conventional tangential field plans in free breathing. Our analysis confirms particularly good cardiac sparing with MCBT-APBI, so that this technique should be offered to patients with left-sided breast cancer if the tumor-associated eligibility criteria are fulfilled.

## Introduction

Breast cancer is the most frequent cancer in women. Due to significant improvements in treatment, long-term survivorship is frequent [1]. Nonetheless, treatment could come with a cost: both, systemic therapies and radiotherapy have an impact on cardiac morbidity [1, 2].

As an example, drug-related cardiac morbidity can be caused by trastuzumab, which is associated with the occurrence of left-ventricular dysfunction and chronic heart failure (CHF) [3, 4]. Well known are also the effects of anthracyclines, causing myocardial damage which can progress to symptomatic CHF. This cardiotoxicity is dose related, progressive, and irreversible [5].

Postoperative radiotherapy for left-sided breast cancer has also an influence on cardiac morbidity. Radiation-related cardiac toxicity is also irreversible and dose dependent. Darby et al. calculated a linear increase in severe cardiac events of 7.4% per gray mean cardiac dose [6]. Radiation-related cardiac toxicity is a late-occurring event, manifesting clinically 10 or more years after breast cancer treatment [7] so that late sequelae observed clinically today reflect radiation techniques used in clinical practice 10 years ago. Moreover, doses to cardiac substructures like the LAD are predictive for defined cardiac events after radiation therapy, as shown for esophageal cancer [8]. With modern treatment techniques, the rate of serious sequelae may be lower than previously thought [9] and several modern radiotherapy techniques significantly improved the therapeutic ratio [10]. This makes it even more important to evaluate the contribution of current radiation techniques to cardiac morbidity in clinical practice. Numerous strategies are available to lower the cardiac dose, such as radiobiologically optimized dosing and fractionation regimes, reduction of target volume, or technical means to distance the heart from the target volume. All appropriate means for heart sparing have to be evaluated against the oncologic risk constellation but also in terms of other patient-related factors like age, individual anatomy, concurrent disease, and locally available techniques to guide individually optimized treatment decisions.

In detail, the following techniques can be considered: deep-inspiration breath-hold (DIBH) is the best-studied technique for heart sparing. Treating exclusively the tumor bed—and thus a smaller volume (as in accelerated partial-breast irradiation, APBI [11])—could also result in better heart sparing. Patients for APBI have to be carefully selected to achieve an equivalent outcome compared to whole-breast radiation therapy [11, 12]. There is some overlap of patients who, on the one hand, can be treated by APBI, and who on the other hand, if treated by WBI, would get a tumor bed boost (e.g. pT2 tumor ≤ 3 cm or patients aged between 45 and 50 years) [12, 13]. The data on APBI for the topic of dose exposure to the heart are, however, not conclusive. A study of Alonso et al. [14] compared heart doses of patients treated with single-catheter intraoperative radiation therapy (IORT) to whole-breast irradiation (WBI) with deep-inspiratory breath-hold. They found that the mean heart dose (Dmean) was significantly lower with DIBH-WBI compared to IORT. Similarly, a study of Dutta et al. [15] found the mean heart dose to be higher in IORT than in DIBH-WBI or DIBH-external beam APBI.

On the other hand, a study on APBI with multicatheter brachytherapy (APBI-MCBT) found a significantly lower dose in the APBI-MCBT group as compared to WBI [16]. Compared to CyberKnife® (CK) radiation, MCBT-APBI performed better in terms of protection of the skin and ribs, whereas CK treatment did show some lower values for non-target breast. The dose parameters for the heart did not differ significantly between the two techniques [17]. Major et al. provide a comprehensive literature review of dosimetric studies between brachytherapy and external-beam radiation therapy including single-fraction boost with BT and VMAT, APBI with MCBT, IMRT, and CyberKnife® (Accuray, CA, USA) [18]. All of the summarized studies show excellent target coverage and sparing of OARs for breast BT. A plan analysis comparing IMRT-APBI and MCBT-APBI showed that the mean dose to the heart was lower with IMRT (2% vs. 4.5%), but as a consequence of IMRT planning, the dose to the lung became larger. Regarded as an absolute value, the MCBT mean heart dose was only 1.3 Gy [19].

We consistently perform both techniques (DIBH and MCBT) for breast cancer patients in our department. The technique is chosen with regard to the ESTRO and ASTRO APBI recommendations [20, 21], respecting the patients’ preferences. The aim of this dosimetric plan analysis is to assess what degree of heart sparing can be achieved in daily routine practice by multicatheter brachytherapy and DIBH.

## Materials and methods

A total of 30 patients treated with multicatheter brachytherapy for left-sided breast cancer were chosen from our database. Twenty-nine of these patients received MCBT as accelerated partial-breast irradiation, one patient (aged 37 years old) received MCBT as boost following external-beam radiation therapy (EBRT). A retrospective EBRT planning approach was done for these patients as described below. These treatment plans were compared to the treatment plans of 22 consecutive left-sided breast cancer patients treated with DIBH radiation therapy. As one could expect, none of the MCBT patients received neoadjuvant chemotherapy, and all fulfilled the treatment criteria for APBI [21].

**Fig. 1:**
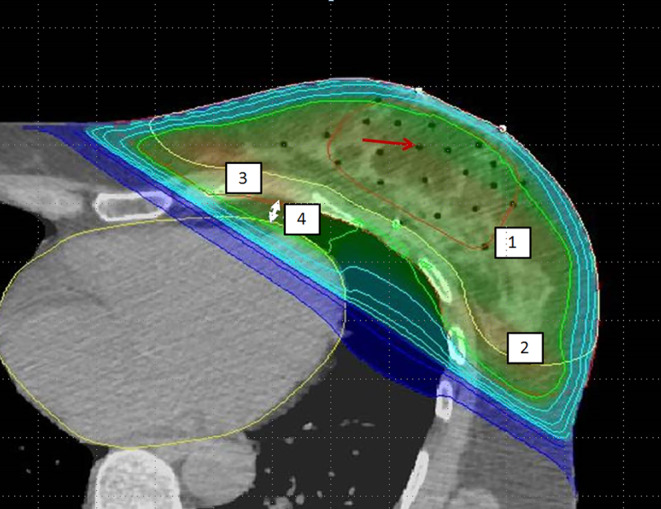
Tangential field treatment plan in a brachytherapy planning CT: *1 *demarks the brachytherapy PTV (*red*); *2* the mammary gland; *3* the EBRT PTV, the *red arrow* shows the air within a brachytherapy single leader catheter; *4* the measure of the PTV–heart distance (*white arrows*) for WB

For the MCBT patients, contouring was performed as described in the paper of Strnad et al*.* [20]. Briefly, in a pre-interventional CT, entry and exit points of the guide needle were localized using in-room lasers. Then, the relevant areas of the surgical scar were implanted with single leader catheters. The PTV safety margin was calculated by considering the size of free resection margins (total size of safety margin was always set to 20 mm), which was the sum of the surgical and added safety margins. The PTV was limited to chest wall/pectoral muscles. The evaluated MCBT dose prescription was 32 Gy in eight fractions administered twice daily for all cases, according to the GEC-ESTRO recommendations [22]. For the dosimetric analysis of this study, the mammary gland CTV (according to the ESTRO contouring guideline [23, 24]) and the heart (according to the Feng et al. atlas [25]) were defined retrospectively on the brachytherapy planning CT (see Fig. 1). Planning target volumes (PTV) were generated by adding a safety margin of 1 cm to the CTV (adapted to natural borders) and were used for external-beam radiotherapy treatment planning in free breathing. The tangential field treatment plans created for this study in free breathing (TF-FB) had a PTV dose prescription of 50 Gy in 2‑Gy single doses (normofractionated whole-breast irradiation, nWBI); 40.05 Gy in 2.67-Gy fractions (moderately hypofractionated WBI, mhWBI), which is seen as today’s standard of care when given without a SIB [26]; and 26 Gy in 5 fractions for the ultrahypofractionated FAST-Forward regimen (uhWBI) [27].

All DIBH patients were treated with a simultaneous integrated boost (SIB) to the primary tumor bed. Dose prescription for the DIBH-WBI PTV was 50.4 Gy in 1.8-Gy and 63 Gy in 2.25-Gy fractions for the SIB [28]. For further comparisons, we calculated tangential field plans (TF-DIBH) without a boost for the treated 22 cases with the standard dosing scheme of 40.05 Gy/15 fractions [13].

Herein, we report the nominal average dose (Dmean) to the heart as well as the mean biologically effective dose (BED) and the mean equivalent dose in 2‑Gy fractions (EQD2) for an α/β of 3 [29, 30].

We additionally analyzed the distance between the outer PTV and the heart contour. For this purpose, the contoured PTV was evaluated slice by slice in the planning CT with a measurement tool, and the minimum measured heart–PTV distance was noted by two independent physicians (see Fig. 1). In case of differences, the respective CT was reviewed simultaneously by both, and differences were clarified. The statistical analyses were performed with IBM SPSS Statistics v. 26 (IMB Corp., Armonk, NY, USA). Correlations between the heart Dmean values were analyzed by *t*-test, correlations between the heart Dmean and the PTV–heart distance by Spearman’s correlation. A value of *p* < 0.05 was considered to be statistically significant.

## Results

Median age of patients treated with MCBT was 63 years (95% CI 57.89–63.78, range 37–76), median age of the patients treated with DIBH was 57 years (95% CI 53.18–60.35, range 41–73). Dmean data are summarized in Table 1 and discussed below.

**Table 1:** Results summary

	MCBT	TF-FB nWBI	TF-FB mhWBI	TF-FB uhWBI	DIBH-SIB	TF-DIBH
Dmean (Gy)	1.28	4.17	3.33	2.12	1.91	1.42
Dmean EQD2 (Gy)	0.81	2.65	2.16	1.47	1.18	0.84
Dmean BED (Gy)	1.35	4.42	3.6	2.45	1.97	1.47

*MCBT* Multicatheter brachytherapy, *TF-FB* tangential field radiation therapy in free breathing, *nWBI* normofractionated whole breast radiation therapy, *mhWBI* moderate hypofractionated whole breast radiation therapy, *uhWBI* ultra hypofractionated whole breast radiation therapy, *DIBH* deep inspiration breath-hold, *SIB* simultaneous integrated boost

### MCBT-treated patients

The heart Dmean for MCBT (for a summed dose of 32 Gy) was 1.28 Gy (95% CI 1.11–1.44, range 0.32–2.0), for TF-FB nWBI was 4.17 Gy (95% CI 3.70–4.63, range 2.16–7.2), for TF-FB mhWBI 3.33 Gy (95% CI 2.96–3.71, range 1.73–5.76), and for TF-FB uhWBI 2.12 Gy (95% CI 1.86–2.38, range 0.92–3.74).

The EQD2 heart Dmean for MCBT was 0.81 Gy (95% CI 0.7–0.92, range 0.19–1.3), for TF-FB nWBI was 2.65 Gy (95% CI 2.34–2.96, range 1.33–4.73) for TF-FB mhWBI 2.16 Gy (95% CI 1.9–2.42, range 1.08–3.9), and for TF-FB uhWBI was 1.47 Gy (95% CI 1.27–1.67, range 0.59–2.8).

The BED heart Dmean for MCBT was 1.35 Gy (95% CI 1.17–1.53, range 0.32–2.17) for TF-FB nWBI was 4.42 Gy (95% CI 3.9–4.94, range 2.22–7.89) for TF-FB mhWBI 3.6 Gy (95% CI 3.17–4.03, range 1.8–6.5) and for TF-FB uhWBI was 2.45 Gy (95% CI 2.11–2.79, range 0.98–4.67).

### DIBH-treated patients

The heart Dmean for DIBH treatment was 1.91 Gy (95% CI 1.67–2.16, range 0.94–2.98), EQD2 Dmean was 1.18 Gy (95% CI 1.02–1.33, range 0.57–1.86), and BED Dmean was 1.97 Gy (95% CI 1.71–2.22, range 0.95–3.10).

Tangential field plans of the DIBH cases (TF-DIBH) with one of today’s most common fractionation schemes of 40.05 Gy in 15 fractions yielded a heart Dmean of 1.42 Gy (95% CI 1.18–1.66, range 0.67–2.46), an EQD2 Dmean of 0.84 Gy (95% CI 0.67–1.02, range 0.09–1.56) and a BED Dmean of 1.47 Gy (95% CI 1.21–1.73, range 0.68–2.59).

**Fig. 2:**
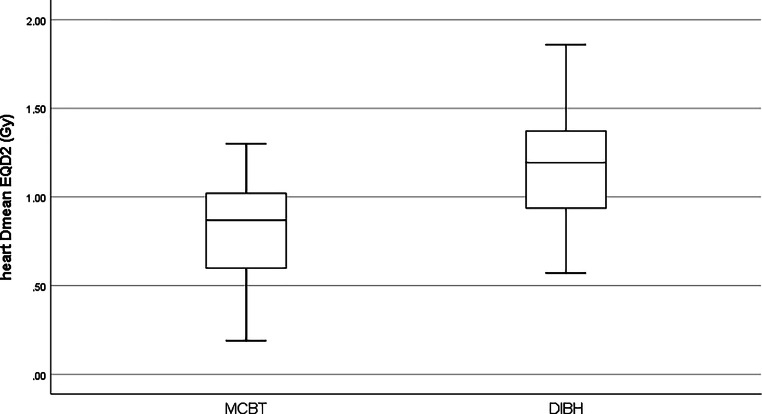
Heart EQD2 Dmean in MCBT and DIBH SIB

Analyzing heart Dmean (numerical, EQD2, and BED) for MCBT and DIBH-SIB patients by *t*-test showed that although the absolute Dmean in each group was very low, they differed significantly. Numerical Dmean was 1.28 Gy vs. 1.91 Gy (*p* < 0.001), EQD2 was 0.81 Gy vs. 1.18 Gy (*p* < 0.001), and BED was 1.35 Gy vs. 1.97 Gy (*p* < 0.001). The boxplot for EQD2 Dmean is shown in Fig. 2. As one could expect, none of the TF-FB plans were able to perform better regarding the heart dose than the DIBH plans, even if using the FAST-Forward regimen: EQD2 Dmean TF-FB uhWBI 1.47 Gy vs. 1.18 Gy EQD2 DIBH (*p* < 0.001).

Statistical analysis by *t*-test of the heart Dmean (numerical, EQD2, and BED) for MCBT and TF-DIBH did not show any significant differences. The calculated values were as follows for MCBT vs. TF-DIBH: Dmean 1.28 vs. 1.42 Gy (*p* = 0.31), Dmean EQD2 0.81 vs. 0.84 Gy (*p* = 0.7), and Dmean BED 1.35 vs. 1.47 Gy (*p* = 0.4). Thus, the most often used hypofractionation scheme achieves doses to the heart as low as those achieved by MCBT. Part of the results of the statistical analysis is shown in Table 2.

**Table 2:** Comparison of heart Dmean values and corresponding *p*-values

Treatment	DIBH-SIB	*p*-value	TF-DIBH	*p*-value
MCBT				
Dmean EQD2 (Gy)	0.81 vs. 1.18	< 0.001	0.81 vs. 0.84	NS
Dmean BED (Gy)	1.35 vs. 1.97	< 0.001	1.35 vs. 1.47	NS

*MCBT* Multicatheter brachytherapy, *DIBH* deep inspiration breath-hold, *SIB* simultaneous integrated boost, *TF* tangential field radiation therapy

**Fig. 3:**
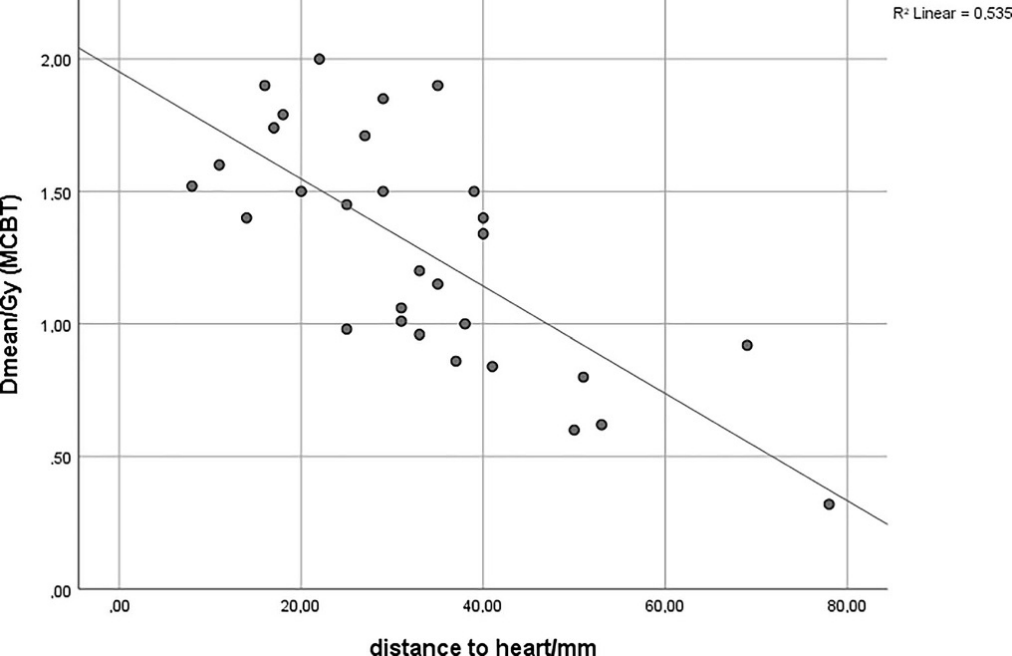
Scatterplot heart distance in mm/heart, Dmean MCBT

The mean heart–PTV distance for MCBT patients was 33.1 mm (95% CI 27.2–39 mm, range 8–78 mm) and for DIBH patients 7.8 mm (95% CI 6.4–9.2 mm, range 3–16 mm), and means differed significantly between these two groups (*p* < 0.001). The heart Dmean for MCBT was significantly associated with the heart-PTV distance (*p* < 0.001), as shown by Spearman’s correlation. The distance–dose distribution for MCBT is shown as a scatterplot in Fig. 3. There was no significant association between the heart-PTV distance with regard to the heart Dmean for DIBH plans (*p* = 0.398).

## Discussion

As we hypothesized, mean heart doses in MCBT radiation therapy were lower than in free-breathing nWBI, mhWBI, and uhWBI, as well as in SIB-DIBH treatments. Hypofractionation translates into a lower dose to the heart, which is a logical consequence of lower nominal total prescription dose. Nonetheless, the ultrahypofractionated regimen of the FAST-Forward trial has not become a standard yet. In 2020, the trial’s 5‑year follow-up data were published. Although with the potential to become a new standard, to date, 15–16 fractions remain the most frequently used hypofractionated regimen. In our retrospective dosimetric analysis, the heart Dmean for the mhWBI treatment plans was 3.33 Gy, which was more than 2.5-fold the dose of the MCBT plans. There is also literature supporting the use of DIBH instead of brachytherapy. The studies of Alsono et al. and Dutta et al. [14, 15] both found better heart sparing by DIBH compared to balloon brachytherapy. The Alonso et al. [14] study comprised 34 patients: 17 patients with left-sided breast cancer treated with a multicatheter balloon in a phase I clinical trial and 17 patients with left-sided tumors who had undergone lumpectomy and adjuvant WBI-DIBH. The mean heart BED was lower with WBI-DIBH as compared to balloon brachytherapy (0.62 vs. 1.3 Gy, *p* = 0.0001). Dutta et al. [15] analyzed 52 consecutive patients with left-sided breast cancer treated with either balloon brachytherapy (*n* = 17; 76% outer breast, Contura Hologic® five-channel balloon), adjuvant external-beam APBI-DIBH (*n* = 18; 56% outer breast, 6% cavity boost), or WBI-DIBH without SIB (*n* = 17, 76% outer breast, 53% with lumpectomy cavity boost). Mean heart BED was higher with balloon brachytherapy, at 1.26 Gy compared to 0.48 Gy and 0.24 Gy for WBI-DIBH and APBI-DIBH, respectively (*p* < 0.001). The results themselves are intriguing, especially as over 75% of patients in the brachytherapy group had a tumor in the outer breast and, thus, it is expected, due to the heart–PTV distance, that the Dmean of the heart would be lower in the APBI-brachytherapy group. Similarly, Holliday et al. found a higher BED to cardiac structures with APBI using single-entry catheter APBI (*n* = 5), Contura® balloon (*n* = 11), and the SAVI® system (*n* = 39) than using DIBH-techniques [31].

However, all these studies used single-entry devices, with some of them capable of modifying the radiation dose distribution (more than one lumen).

Multicatheter brachytherapy—which can better modulate the dose to the PTV and thus significantly increase dose conformity—should be expected to perform better with regard to heart sparing.

Lettmaier et al. [16] found a significantly lower radiation exposure to all organs at risk using MCBT-APBI. They created two physical treatment plans for each of 16 patients with left-sided breast cancer, one for sole external-beam radiotherapy and one for partial-breast brachytherapy using MCBT. The exposed dose to a prespecified volume (D0.5cc, D1cc, up to D50cc) of the heart was significantly lower using MCBT than WBI, with D0.5cc being 11.82 Gy vs. 44.06 y, D1cc being 10.72 Gy vs. 41.91 Gy, and D50cc being 5.6 Gy vs. 18.17 Gy.

The smaller, nontangential PTV in PBI often results in a longer distance to the heart compared to WBI. In our cohort, the PTV to heart distance in DIBH patients was 7.8 mm, which is a result of the target definition process, because the adjacent thoracic wall is an integral part of the PTV. During beam-on time in DIBH radiation therapy, the PTV to heart distance should be reproducible. MCBT planning CTs were done in free breathing. It is possible that the PTV to heart distance that was measured shrinks during the patient’s exhale phase, and that the real heart dose is somewhat higher. It should be pointed out the dose to the LAD could differ, because the heart Dmean is not a perfect surrogate parameter for it, but this is beyond the scope of this article [32]. In principle, it is feasible to apply APBI in DIBH. MCBT-APBI is not suitable for every breast cancer patient [21]; nevertheless, there is some overlap of patients that can be treated by sole ABPI, or, if treated by WBI, would get a tumor bed boost [12, 13]. The reason for the discrepancy regarding results using APBI-brachytherapy in the published literature is the outcome of the different techniques that are used for APBI. Single-entry devices have no or few possibilities for 3D-optimized dose distribution, whereas MCBT offers the complete armamentarium of modern radiation planning and dose optimization. Patients treated with neoadjuvant chemotherapy do not qualify for APBI, so for these patients, DIBH offers the possibility of whole-breast radiation with low doses to the heart, also using an SIB, when indicated. Only some specialized centers are equipped and experienced enough to offer MCBT, but these should offer MCBT-APBI to suitable patients as an alternative to WBI.

There are of course several limitations to our study. The TF-FB planning was performed retrospectively on the MCBT CT datasets. But even if the mammary gland is slightly compressed by the brachytherapy catheters, an anatomic shift of the bony thorax is unlikely, and we thus considered the treatment plans similar to daily FB routine treatments. We further chose this approach (retrospectively planning WBI on MCBT CTs) in order to have a fair comparison to the MCBT dose that is delivered in FB. In most departments, DIBH is standard for left-sided WBI, and our retrospective plan analysis showed that this technique can achieve comparably low doses to the heart, despite the SIB. It is important to note that in our analysis, the heart was retrospectively contoured in the MCBT plans and that no dose optimization to the cardiac structures was performed during MCBT treatment planning. Optimizing for specific heart constraints would probably result in even lower doses than those presented herein for the MCBT treatment plans. On the other hand, normofractionated DIBH plans were optimized to the heart structures and, although using an SIB concept, reached low doses of 1.91 Gy heart Dmean. Further, as shown by the calculation of hypofractionation TF-DIBH plans, DIBH will result in very low doses. A hypofractionted regimen is now carried out as a daily routine. It should be noted that the combination of hypofractionation and a boost given as SIB, which could also provide some advantage for sparing radiation dose to the heart, but is currently seen as an experimental therapy per German S3 guideline [13].

## Conclusion

Both MCBT-APBI and DIBH using an SIB can lead to low doses to the heart and, thus, may have an impact on cardiac morbidity. This may be even more relevant as the subgroup suited for MCBT-APBI in general shows good prognostic characteristics. On the basis of an informed-consent decision process, MCBT-APBI carried out at experienced centers should be offered to left-sided breast cancer patients who fulfill the eligibility criteria as one possible treatment modality. Without optimizing the dose to the heart during the planning process, results as low as with DIBH radiation therapy can be achieved.
